# Perception of Dental Faculty Regarding Development of Preclinical Endodontic Simulation Curriculum for Undergraduate Dental Students in Pakistan

**DOI:** 10.1155/sci5/1988107

**Published:** 2026-01-09

**Authors:** Salima Naveed Manji, Muhammad Imtiaz, Saroosh Ehsan, Khizer Mehmood, Shahzad Ahmad, Naauman Zaheer, Shazia Iqbal, Shahzad Ali

**Affiliations:** ^1^ Department of Dental Education, FMH College of Medicine and Dentistry, Lahore, Pakistan; ^2^ Department of Oral Maxillofacial Surgery, FMH College of Medicine and Dentistry, Lahore, Pakistan; ^3^ Department of Operative Dentistry, FMH College of Medicine and Dentistry Lahore, Lahore, Pakistan; ^4^ Department of Prosthodontics, Azra Naheed Dental College, Lahore, Pakistan; ^5^ Faculty of Medicine and Health Science, The University of Buckingham, Buckingham, UK; ^6^ Department of Oral Biology & Tooth Morphology, Institute of Dentistry, CMH Lahore Medical College, NUMS, Lahore, Pakistan; ^7^ Department of Pharmacy and Biotechnology, Alma Mater Studiorum - Università di Bologna, Bologna, Italy; ^8^ Department of Information Sciences, University of Education, Lahore, Pakistan, uew.edu.gh

**Keywords:** assessment, endodontics, haptic virtual reality, patient safety, simulated teaching

## Abstract

**Introduction:**

Dental education’s globalization necessitates that curricula meet international standards, and simulation technologies provide opportunities to enhance skill acquisition in endodontics. This study investigates faculty perceptions on implementing a simulation‐based curriculum in Pakistan, focusing on preclinical training effectiveness and potential integration challenges.

**Methods:**

A cross‐sectional study, employing probability sampling, was conducted among endodontics faculty across Pakistan. Using a validated online questionnaire distributed via Google Forms, the study gathered quantitative data on faculty perceptions regarding the simulation curriculum’s design, assessment methods, and implementation.

**Results:**

Among the 33 participants, 63.6% were male, and 69.7% had over 8 years of experience. Faculty responses indicated strong support for simulation integration, with concerns about feasibility given resource limitations. Many endorsed haptic VR’s potential for improving clinical skills, though practical barriers such as cost and faculty training were highlighted.

**Conclusion:**

There is significant interest in implementing a structured simulation‐based endodontics curriculum in Pakistan. While faculty are positive about simulation’s benefits, institutional support and resource allocation will be essential for effective integration to enhance skill acquisition and patient safety in endodontics education, ensuring that students receive comprehensive training and workshops that meet international standards. By leveraging simulation‐based learning, dental education in Pakistan can evolve to produce competent and confident practitioners equipped to address the challenges of modern clinical practice.

## 1. Introduction

The globalization of dental education and the demand for international standardization have fostered significant advancements in dental curricula worldwide [1]. Technological innovations, such as dental haptic virtual reality (VR) simulation systems, have been integrated into many dental programs to enhance students’ clinical skills in a controlled, risk‐free environment [2, 3]. Endodontics, a cornerstone of clinical dental practice, benefits greatly from such simulation‐based training, which allows students to develop critical skills without compromising patient safety [4–6]. In this context, simulation‐based learning provides an effective approach for achieving skill acquisition and proficiency, especially in resource‐limited regions like Pakistan, where access to sophisticated training facilities may be constrained.

Dental simulation, in particular, offers tactile feedback and enables students to experience realistic clinical scenarios [7, 8]. This technology supports the motor learning process, guiding students through stages from basic skill acquisition to automated, complex procedures. The cognitive, associative, and autonomous stages of motor skill learning, as described by Fitts and Posner, are applicable to dental training, where repeated, deliberate practice is key to skill mastery. Despite the promise of simulation in dental education, the cost, accessibility, and acceptance of this technology in developing countries remain significant barriers [9, 10].

In Pakistan, the preclinical years in dentistry play a crucial role in laying the foundation for clinical training; therefore, incorporating endodontic simulation during this phase can help minimize errors once students begin treating patients in their clinical years. The need for curriculum reform is underscored by the gap between the existing methods and the competencies required to meet modern dental practice standards. The current research focuses on the perceptions of dental faculty regarding the development and implementation of a preclinical endodontic simulation curriculum for undergraduate dental students. By assessing faculty views on curriculum structure, content, and potential outcomes, this study aims to inform the design and adoption of a standardized, simulation‐based endodontic preclinical curriculum that aligns with international best practices and prepares students for real‐world challenges in dental care [11–13].

## 2. Problem Statement

The field of dental education is evolving globally, with simulation‐based learning becoming integral to preclinical as well as clinical training [14]. In endodontics, simulation enables students to develop essential hands‐on skills while safeguarding patient safety [15]. However, in Pakistan, the curriculum for preclinical endodontics has lagged behind due to limited or no access to modern simulation technologies, such as dental haptic VR systems, and the lack of standardized simulation‐based curricula [12, 15, 16]. Traditional teaching methods in Pakistan primarily rely on didactic instruction and limited practical exposure, which may not fully prepare students for the complexities of clinical practice. This gap highlights the need to reassess and reform the current educational approach to align with international standards and enhance competency in endodontics for undergraduate students.

## 3. Research Gap

While many countries have embraced dental simulation, which provides immersive, tactile learning experiences, the adoption of these technologies in Pakistani dental institutions remains sparse. Few studies have evaluated the effectiveness or feasibility of integrating simulation‐based endodontic curricula in Pakistan [13, 17, 18]. Moreover, there is limited research exploring the perceptions of Pakistani dental faculty on this topic. This study aims to address this gap by investigating the perspectives of endodontics faculty in Pakistan on the development and potential implementation of a simulation‐based curriculum, focusing on its perceived benefits, challenges, and required resources [19–21].

## 4. Research Question

What are the perceptions of dental faculty in Pakistan regarding the development and implementation of a preclinical endodontic simulation curriculum for undergraduate dental students?

## 5. Hooking Point

As dental education in Pakistan strives to meet global standards, the inclusion of simulation‐based learning, particularly in critical areas like endodontics, is not just beneficial but essential. Modern simulation tools, such as dental haptic VR, have the potential to transform the training landscape by providing realistic, hands‐on experiences that enhance skill acquisition and reduce clinical errors. However, the views of faculty, who play a crucial role in curriculum development and implementation, are vital to understanding the feasibility and value of such advancements in the local context. Exploring these insights can provide actionable data to drive meaningful reforms in dental education in Pakistan.

## 6. Rationale

The integration of simulation‐based learning into dental curricula has shown promising results worldwide, improving students’ clinical skills, patient safety awareness, and overall competency. Given the limited exposure to modern simulation technologies in Pakistan’s dental institutions, there is a pressing need to assess whether faculty members are open to such changes and what specific challenges they foresee. By focusing on faculty perceptions, this study seeks to identify both the potential benefits and the obstacles to implementing a standardized, simulation‐based curriculum. These insights can aid in developing a more effective and comprehensive endodontics training program that better prepares Pakistani dental students for clinical practice, ultimately contributing to improved dental care in the country.

## 7. Aim

To explore the perceptions of dental faculty in Pakistan regarding the need for and development of a preclinical endodontic simulation curriculum for undergraduate students, with a focus on understanding the perceived benefits, challenges, and requirements for successful integration of simulation‐based learning.

## 8. Objectives

1. To assess faculty perceptions of the current endodontic curriculum and its effectiveness in preparing students for clinical practice.
2. To explore faculty views on the potential benefits of incorporating simulation‐based learning into preclinical endodontic training.
3. To identify perceived challenges and barriers to the implementation of a simulation‐based curriculum in Pakistani dental institutions, including resource limitations and faculty training requirements.
4. To gather faculty recommendations on curriculum design, content, and assessment methods for a standardized, simulation‐based preclinical endodontics curriculum aligned with international standards.

This study aims to provide data‐driven recommendations for curriculum developers, decision‐makers, and policymakers in the dental education sector, supporting efforts to modernize and enhance endodontic training in Pakistan.

## 9. Methodology

The survey tool was adapted with permission and approval from the original author [12]. Perry identified current patterns in the role and integration of simulation in dental degree curricula internationally by distributing a survey to clinical curriculum leaders in dental schools in Asia, Europe, North America, and Oceania (Australia and New Zealand).

This study employs a cross‐sectional quantitative research design to examine the perceptions of endodontics faculty across Pakistan on the development of a simulation‐based preclinical curriculum in endodontics. Responses were collected and analyzed using statistical software. Descriptive statistics were used to analyze demographic data and summarize faculty responses. Further analysis was conducted to explore trends and correlations between faculty perceptions and variables such as years of experience and academic rank.

Ethical approval for the study was obtained from the Institutional Review Board (IRB) of Fatima Memorial College of Medicine & Dentistry Lahore (approval number: FMH‐03‐2021‐IRB‐881‐M). Participation was voluntary, and consent was obtained from all participants prior to data collection.

This methodology provides a structured approach for understanding the perspectives of endodontics faculty on the integration of simulation‐based learning into the dental curriculum, addressing both the opportunities and challenges of implementing such innovative training methods in Pakistan.

## 10. Purposive Convenience Criterion Sampling Was Done

**Sample unit:** Participants: Faculty participatory approach (endodontics department Prof, Assoc. Prof, Assist. Prof).

**Inclusive criteria:** Those who are working in the endodontics department and have more than 3 years of teaching experience in endodontics and know‐how regarding simulation curriculum.

**Exclusive criteria:** Those faculty members who have less experience and belong specifically to the operative side rather than endodontics.

**Impact of the study:** This needs analysis will serve as a guide for the curriculum developers. And they may incorporate this need analysis into developing future dental curriculum. And this will further help in getting students’ feedback related to the dental simulation curriculum. As a result, through this it is expected that through simulation curriculum learning outcomes and patient care may improve through early endodontics hands‐on exposure and expertise.

## 11. Study Population and Sampling

The study targets faculty members specializing in endodontics from various dental colleges across Pakistan. Participants are selected using a purposive convenience criterion sampling method to ensure that the sample is representative of the diverse faculty demographic. Inclusion criteria include dental faculty with experience teaching endodontics, while faculty from other dental specialties are excluded. A total of 33 faculty members participated in the study, representing a mix of academic ranks and professional backgrounds. The demographic summary of the study population is presented in Table 1.

**Table 1:** Demographic summary of the study cohort.

Variable	Description
Samples (n)	33

Age (years)	38.5 ± 10.8 (range: 0–67)

Gender	Male: 21 (63.6%)
	Female: 12 (36.4%)

Designation	Professor: 11 (33.3%)
	Associate professor: 4 (12.1%)
	Assistant professor: 11 (33.3%)
	Resident: 5 (15.2%)
	Private practitioner: 1 (3.0%)
	In charge of simulation: 1 (3.0%)

Experience (years)	> 8 years: 23 (69.7%)
	5–6 years: 5 (15.2%)
	7–8 years: 2 (6.1%)
	3–4 years: 2 (6.1%)
	1–2 years: 1 (3.0%)

## 12. Data Collection

Data were collected through a validated online questionnaire administered via Google Forms. The questionnaire was designed to capture comprehensive insights into faculty perceptions regarding the need for a simulation curriculum, general attitudes toward simulated teaching, and their views on the assessment, evaluation, and implementation of such a curriculum. To ensure reliability and validity, the survey instrument underwent expert review and a pilot test.

## 13. Results

The study gathered responses from 33 endodontics faculty members from various dental colleges across Pakistan. The total number of participants in this study included 21 (63.6%) males and 12 (36.4%) females. 69.7% of the participants had more than 8 years of experience; the remaining 30.2% had more than 2 years of experience, respectively. The participants were given a consent form along with the questionnaire to assess their perception regarding the preclinical endodontology simulation curriculum for undergraduate dental students. This extensive experience suggests that the participants possess a solid foundation in endodontics education, which provides valuable insights into their perspectives on curriculum reform. The survey results are presented below in graphical form, highlighting different areas. The data about the designation of the participants are shown in Table 2.

**Table 2:** Faculty perception on adequacy of preclinical endodontics training.

Designation	Needs improvement	No	Yes	Total
Professor	1 (9.1%)	**8** (72.7%)	2 (18.2%)	11
Associate professor	0 (0.0%)	1 (25.0%)	3 (75.0%)	4
Assistant professor	0 (0.0%)	**9** (81.8%)	2 (18.2%)	11
Resident	0 (0.0%)	1 (20.0%)	**4** (80.0%)	5
Private practitioner	0 (0.0%)	1 (100.0%)	0 (0.0%)	1
In charge of simulation	0 (0.0%)	0 (0.0%)	1 (100.0%)	1
Total	1	20	12	33

## 14. Summary of Findings

The data reveal that endodontics faculty in Pakistan are generally supportive of integrating simulation‐based learning into the undergraduate curriculum. They recognize the potential benefits for skill acquisition and patient safety but also acknowledge the financial and logistical hurdles that must be overcome. As the faculty expressed a willingness to adapt to new teaching methods, their insights could serve as a foundation for advocating the adoption of dental simulation technology in Pakistani institutions. Overall, these findings suggest that with targeted support and strategic planning, simulation‐based training could play a transformative role in advancing dental education in Pakistan.

The faculty perceptions of preclinical training adequacy varied by designation.

• **Professors:** 72.7% (8/11), rated the training as inadequate, while 18.2% (2/11) found it adequate.
• **Associate professors** were more positive, with 75.0% (3/4) rating the training as adequate.
• **Assistant professors** expressed the highest dissatisfaction, with 81.8% (9/11) rating the training as inadequate.

There was no statistically significant association between faculty designation and their perception of the adequacy of preclinical training (chi‐square statistic (χ^2^) = 13.88, *p* = 0.179) (Table 2).

**Figure 1:**
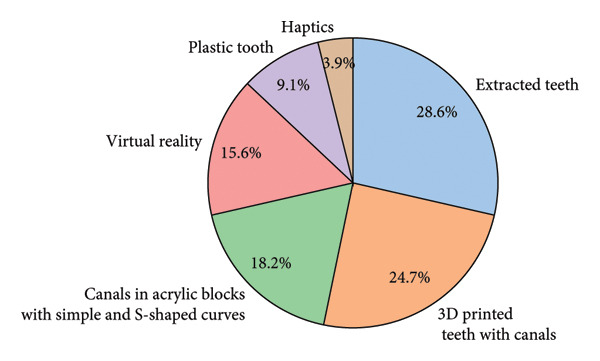
Recommended simulation training methods for preclinical endodontics.

The preferred simulation tools for undergraduate endodontics training were extracted teeth, which were the most recommended tool with 28.6%, followed by 3D‐printed teeth with 24.7% (Figure 1).

**Figure 2:**
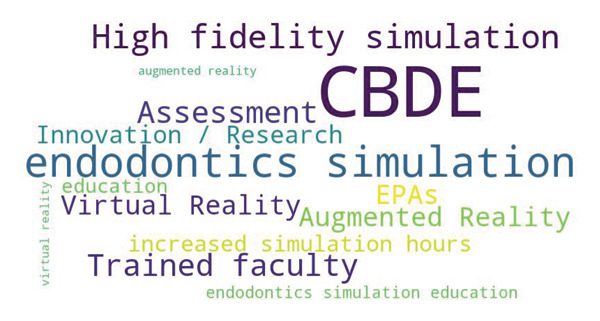
Word cloud plot representing the changes expected for endodontics simulation education in Pakistan in the next 20 years.

Furthermore, the word cloud plot (Figure 2) illustrates the frequency and prominence of key terms provided by faculty members representing the expected changes for endodontics simulation education in Pakistan in the next 20 years. The size of each word corresponds to its frequency. The most prominent (bigger font size) terms are

• Competency‐based dental education (CBDE),
• Trained faculty,
• Endodontics simulation,
• High‐fidelity simulations are also prominently displayed, indicating their relevance to the discussion.

Less frequent (smaller font size) terms were as follows:

• VR/augmented reality,
• Assessment,
• Innovation/research in endodontics simulation,
• EPAs.

**Figure 3:**
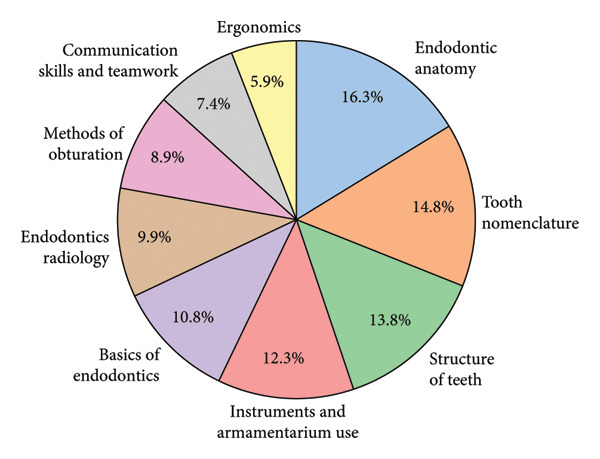
Distribution of key topics mentioned in faculty responses.

All 33 faculty members agree that “Endodontic anatomy” should be included in the theoretical endodontics simulation syllabi. The frequency of key topics mentioned by faculty members is shown in Figure 3. The chart demonstrates that “Endodontic anatomy” (33 mentions) was the most frequent topic mentioned by faculty members, while ergonomics was the least frequent with 12 mentions.

The majority of respondents who have a simulation curriculum (13 respondents: Yes‐Yes) also include competencies in the study guide, suggesting a positive trend between the two variables. However, the presence of No‐Yes (11 respondents) and Yes‐No (4 respondents) indicates that the relationship is not strictly dependent (Table 3).

**Table 3:** Relationship between simulation curriculum and competencies mentioned.

Preclinical endodontics simulation curriculum (Simulation_Curriculum)	Endodontic‐related competencies mentioned in the study guide (Competencies_Mentioned)		Total
	NO	YES	
NO	6	11	17
YES	3	13	16
Total	9	24	33

Furthermore, the significant test (with chi‐square statistic = 1.406 and *p* = 0.236) indicates that there is no statistically significant relationship between the preclinical endodontics simulation curriculum and related competencies mentioned in the study guide, which aligns with the observations from the contingency Table 3.

**Figure 4:**
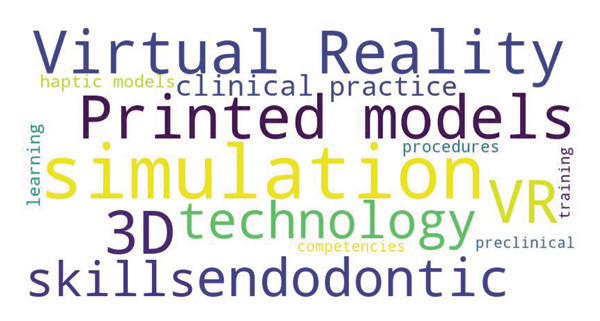
Key themes in simulation‐based endodontic training.

The key terms suggested by respondents to recommend specific simulation technologies (e.g., VR, 3D printed models, and haptic models) and strategies for integrating simulation into the curriculum (Figure 4).

### 14.1. Teaching and Learning

**Figure 5:**
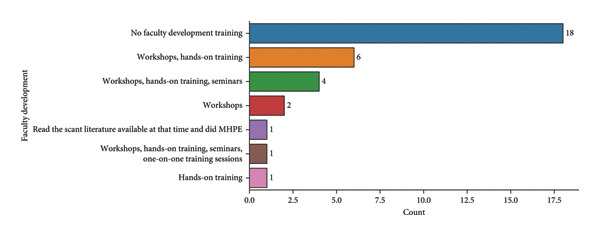
Faculty development for preclinical endodontics simulation.

Figure 5 illustrates various approaches used for faculty development in preclinical endodontics simulation. The majority of faculty members reported receiving no formal development training, followed by those who participated in workshops and hands‐on training sessions. Fewer respondents indicated engagement in seminars or self‐directed learning through literature. This suggests a significant gap in structured faculty training for preclinical endodontics simulation in the phantom lab.

**Figure 6:**
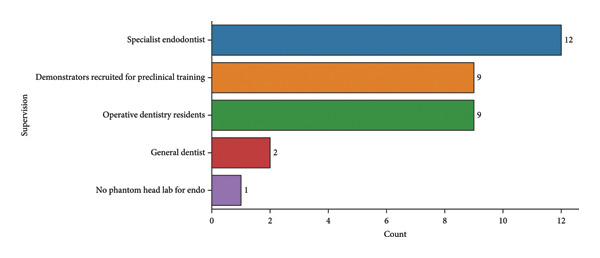
Supervision during preclinical endodontics simulation.

As illustrated in Figure 6, the majority of preclinical endodontic simulation sessions were supervised by specialist endodontists (*n* = 12), followed by demonstrators recruited for preclinical training (*n* = 9) and operative dentistry residents (*n* = 9), while only a small proportion involved general dentists (*n* = 2) or institutions without a phantom head laboratory for endodontic training (*n* = 1). This distribution highlights disparities in specialist supervision and resources, emphasizing the urgent need for faculty development and infrastructural support to standardize preclinical endodontic simulation training in Pakistan.

The mean number of hours allocated per week for conducting preclinical endodontic training varied by designation. Associate professors reported the highest allocation (16 h/week), while private practitioners reported the lowest (5 h/week) (Table 4).

**Table 4:** Mean number of hours allocated to conduct preclinical endodontics training by faculty designation.

Designation	Hours are allocated to conduct preclinical endodontics training (hours/week)
Professor	15
Associate professor	16
Assistant professor	14
Resident	10
In charge of simulation	10
Private practitioner	5

The most frequent root canal preparation method reported was the **step-back technique** (25), followed by the **crown-down technique** (5). In addition, irrigant method used in preclinical endodontic training reported was **Saline** (13 out of 33) (Table 5).

**Table 5:** Methods for root canal preparation and irrigant used in preclinical endodontic training.

Methods of root canal preparation	Count	Irrigant used in preclinical endodontic training	Count
Step back technique	25	Saline	13
Crown down technique	5	Sodium hypochlorite	9
Combination	1	Water	8
Combination of crown down and step back	1	We teach and demonstrate only	1

### 14.2. Assessment

**Figure 7:**
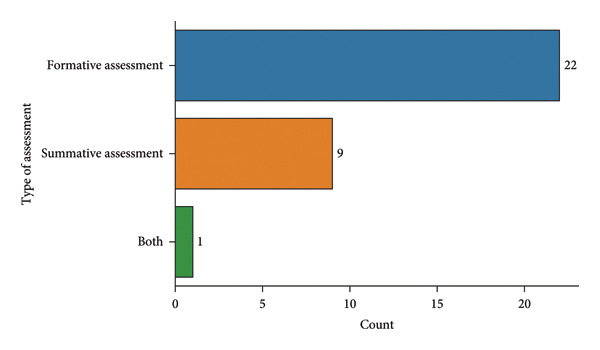
Type of assessment during preclinical endodontic simulation training.

Figure 7 illustrates the distribution of “type of assessment” done during preclinical endodontic simulation training in your institute. **Formative assessment** is the most common type of assessment (22 respondents), followed by **summative assessment** (9 respondents). In addition, a smaller number of respondents use both formative and summative assessments (1 respondent).

In addition, Table 6 shows the methods used to assess preclinical students during endodontic simulation training in the institute. This indicates that the **grading method** is the most frequently used assessment method. The rubric method is also commonly used, followed by pass/fail.

**Table 6:** Assessment methods by faculty designation.

Faculty by designation	Through which method do you assess preclinical students during endodontic simulation training in your institute?				
	Grading method (%)	Rubric method (%)	Grading method: pass/fail method (%)	Grading method, rubric method (%)	Pass/fail method, rubric method (%)
Professor	30	15	0	0	5
Associate professor	12.5	37.5	0	0	0
Assistant professor	36.4	9.1	0	4.5	0
Resident	40	0	10	0	0
In charge of simulation	50	0	0	0	0
Private practitioner	50	0	0	0	0

### 14.3. Evaluation

A majority of respondents (24) reported that the preclinical endodontic simulation curriculum is evaluated, while 18 believed it helps to achieve learning outcomes by course end. Evaluation methods primarily included student feedback (*n* = 19), structured forms (*n* = 8), and a combination of both (*n* = 3), although one respondent reported no evaluation conducted yet (Table 7). The use of simulation tools such as ***VR*** during the course facilitates successfully obtaining the learning outcomes by the end of the course (59.1%) as compared to non‐VR/3D users (54.5%) not being able to achieve all the learning outcomes by the end of the course (Table 8).

**Table 7:** Evaluation of preclinical simulation curriculum.

Evaluation	Yes	No	Other
Achieve learning outcomes by the end of the course?	18	14	1
Evaluate the endodontics preclinical simulation curriculum?	24	8	1
Evaluation methods:	Feedback: 19		
	Forms: 8		
	Forms, feedback: 3		
	Have not done yet: 1		

**Table 8:** Learning outcomes by tool usage.

Do you achieve all your learning outcomes by the end of the course?	Tools usage	
	VR/3D users (%)	Non‐VR/3D users (%)
No	36.4	54.5
Yes	59.1	45.5
Yes, No	4.5	0

### 14.4. Implementation

**Figure 8:**
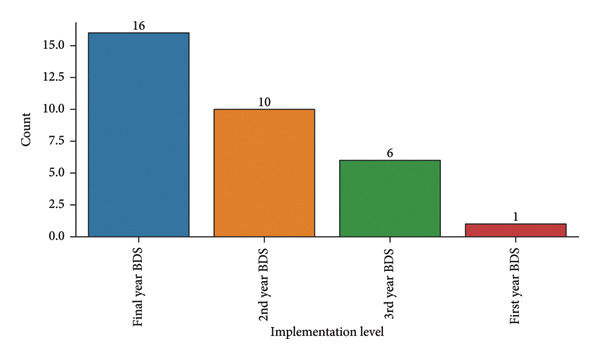
Implementation level of simulated endodontics curriculum.

**Figure 9:**
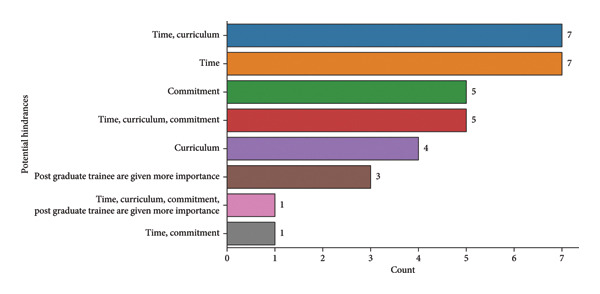
Potential hindrances.

The simulated endodontics curriculum was implemented at various undergraduate levels (Figure 8). Time constraints and curriculum overload were the most commonly reported barriers to effective simulation training (Figure 9). Untrained faculty cited more hindrances, particularly time (*n* = 17) and curriculum‐related issues (*n* = 12), than trained faculty (Table 9).

**Table 9:** Potential hindrances with respect to faculty training.

Potential hindrances	Faculty training	
	Trained	Untrained
Commitment	3	9
Curriculum	5	12
Postgraduate trainee are given more importance	1	3
Time	4	17

## 15. Faculty Rank and Its Impact on Perception

Participants represented a variety of academic positions, with professors and assistant professors each accounting for 33.3%, followed by associate professors at 12.1%, residents at 15.2%, and an additional 6.1% categorized as “others.” This distribution reflects a broad spectrum of professional roles, allowing for a diverse range of opinions and experiences to inform the results. Interestingly, the data suggest that more senior faculty members, such as professors and associate professors, generally expressed a higher level of support for a structured, simulation‐based endodontics curriculum. They also acknowledged the potential benefits of simulation in developing hands‐on skills and enhancing patient safety.

## 16. Perceived Need for Simulation‐Based Curriculum

A large majority of respondents indicated a strong need for the incorporation of simulation technology into the undergraduate endodontics curriculum. Faculty members highlighted simulation as a valuable tool for providing a safe and controlled environment where students can gain procedural experience before performing actual clinical work. This is particularly relevant in a setting like Pakistan, where access to diverse patient cases is immense, but due to socioeconomic factors, institutional resources are limited. The faculty noted that simulation not only enhances technical skills but also fosters critical thinking and decision‐making, essential for a well‐rounded dental practitioner.

### 16.1. Attitudes Toward Haptic VR Technology

While there was enthusiasm about the potential of VR haptic technology, responses were mixed regarding its practicality and feasibility. Many faculty members recognized the technology’s ability to simulate real‐life tactile feedback, which they believed would accelerate students’ motor skill development and improve their transition to clinical practice. However, some faculty members expressed concerns about the cost and accessibility of such technology, noting that budget constraints and lack of institutional resources might hinder its adoption in Pakistan. Despite these reservations, the overall perception was positive, with many participants agreeing that haptic VR could play a pivotal role in modernizing the endodontics curriculum if financial and logistical challenges could be addressed.

### 16.2. Assessment, Evaluation, and Implementation

Regarding assessment methods for a simulation‐based curriculum, the majority of faculty members emphasized the importance of frequent, structured assessments to track student progress. Simulation was seen as particularly effective for competency‐based assessments, which could help ensure that students meet specific skill benchmarks before advancing. Faculty responses also indicated a preference for incorporating debriefing sessions, where students receive feedback on their performance in simulated scenarios. These debriefing sessions were seen as essential for helping students reflect on their learning experiences and identify areas for improvement.

When discussing the practical aspects of curriculum implementation, faculty members acknowledged challenges such as the need for trained faculty, the establishment of dedicated simulation labs, and the development of standardized assessment protocols. Despite these challenges, the consensus was that with adequate planning and investment, a simulation‐based endodontics curriculum could significantly enhance dental education in Pakistan.

## 17. Discussion

This study revealed strong support among Pakistani dental faculty for the integration of simulation‐based learning into the preclinical endodontics curriculum. The participants acknowledged the potential of simulation, especially haptic VR and 3D modeling, to enhance skill acquisition and improve patient safety. These findings are consistent with international literature emphasizing the pedagogical advantages of simulation in dental education [19].

Internationally, simulation‐based education has become an integral part of preclinical dental training [22, 23]. In Europe and North America, institutions employ high‐fidelity simulators and VR platforms to offer structured, competency‐based training in endodontics [12, 24]. For instance, the Simodont Dental Trainer is widely used across European dental schools to bridge the gap between theory and clinical experience, showing evidence of improved psychomotor skills and student confidence [25]. Such tools enable experiential learning through repetitive practice, debriefing, and formative assessment, aligning with adult learning principles and Kolb’s experiential learning theory [12]. In the Pakistani context, however, simulation technologies remain underutilized. Faculty feedback from this study highlighted inconsistencies in simulation implementation, inadequate institutional infrastructure, and the absence of standardized curricular guidelines. These observations resonate with previous local studies, which have criticized the didactic‐heavy and skill‐light nature of dental curricula in Pakistan [26]. The lack of summative assessments and structured documentation in many institutions weakens the capacity to measure learning outcomes effectively, an issue contrasting with the rigor found in international programs [27, 28].

Notably, the majority of Pakistani faculty participants acknowledged the value of simulation in enhancing procedural competency, especially when introduced in the preclinical years. This sentiment supports prior research suggesting that early exposure to realistic procedural tasks helps reduce clinical errors and boosts student confidence before they treat real patients [29, 30]. Faculty also emphasized the necessity of including subjects like endodontic anatomy, ergonomics, and infection control within the simulation syllabi, echoing calls for integrated, clinically relevant content [30].

Despite the enthusiasm, critical barriers were identified, chief among them being time constraints, curriculum overload, and insufficient faculty training. The study’s finding that untrained faculty reported more hindrances mirrors earlier literature emphasizing the importance of structured faculty development to ensure the effective use of simulation tools [31]. Globally, continuing professional development (CPD) modules on simulation pedagogy are standard practice, but such initiatives are sporadic or nonexistent in most Pakistani dental institutions.

The divergence between faculty aspirations and institutional realities underscores the urgent need for policy reform. Regulatory bodies like the Pakistan Medical and Dental Council (PMDC) must play a proactive role in mandating simulation‐based teaching and accrediting simulation labs as part of dental program evaluations. Lessons can be drawn from the Commission on Dental Accreditation (CODA) in the United States, which enforces the inclusion of simulation in preclinical curricula as a quality benchmark [32].

Moreover, the positive outlook of faculty toward simulation over the next 20 years evident in frequent mentions of “competency‐based education,” “haptic feedback,” and “trained faculty” indicates a readiness for cultural and pedagogical transformation. Leveraging this momentum will require collaborative curriculum reform, sustainable investment in infrastructure, and faculty capacity‐building programs at a national level.

## 18. Conclusion

The findings highlight the critical need for a standardized preclinical endodontic simulation curriculum in Pakistani dental institutions. The current lack of summative assessments and a well‐defined curriculum undermines the potential of simulation‐based learning to enhance student competencies and patient safety. By adopting best practices from international universities, such as competency‐based frameworks, high‐fidelity simulations, and structured faculty development programs, Pakistani institutions can bridge the gap and align their dental education with global standards.

To achieve this, the following recommendations are proposed:

1. Developing a standardized curriculum: Institutions should establish a unified curriculum for preclinical endodontic simulation, including defined learning outcomes, assessment protocols, and minimum competency requirements.
2. Introducing summative assessments: Incorporate regular, structured summative assessments to evaluate student performance and readiness for clinical practice.
3. Investing in faculty development: Implement comprehensive training programs to prepare educators for teaching with simulation technologies.
4. Enhancing simulation facilities: Upgrade simulation labs to include high‐fidelity models, haptic VR systems, and other advanced tools to improve training quality.
5. Policy and accreditation reforms: Regulatory bodies like the PMDC should mandate simulation‐based training as a core component of dental education, with periodic audits to ensure compliance.

In conclusion, integrating simulation‐based learning into preclinical endodontics education in Pakistan offers a promising avenue to improve the quality of dental training. However, this requires a concerted effort from institutions, policymakers, and faculty to address resource limitations, standardize curricula, and ensure alignment with international benchmarks.

## 19. Recommendations

The second phase of this research should involve a qualitative advocacy study using one‐on‐one interviews. These interviews will be conducted based on a predesigned template of questions, followed by thematic analysis to further explore faculty perceptions regarding the Endodontics Simulation Curriculum. Insights from the interviews and existing literature will guide the analysis. Subsequently, content validation of the findings will be carried out using the Delphi technique.

Deliberate curricular planning is necessary to reform dental student training. A new standard of care should be implemented to align simulation‐based training with international benchmarks. Ad hoc approaches to curriculum development must be replaced with a well‐defined policy vision, which should be clearly documented and disseminated prior to the implementation of new dental curricula.

## 20. Future Study

Undertake students’ perception as well along with in‐depth interviews of faculty to design a comprehensive simulation curriculum and to identify practical strategies for successfully implementing updated standards.

## 21. Limitations

While this study offers valuable insights, its generalizability is limited by the relatively small sample size and exclusive focus on faculty perceptions. Future research should include dental students to triangulate findings and better understand the impact of simulation on learner outcomes. Furthermore, qualitative investigations using Delphi or thematic analysis can enrich the understanding of contextual challenges and inform the development of a robust, evidence‐based curriculum [33].

## 22. Strengths

The involvement of senior faculty provided critical insights and ensured that the research objectives were effectively addressed.

## Ethics Statement

This study was reviewed and approved by the Institutional Review Board (IRB) of FMH College of Medicine & Dentistry, Lahore, Pakistan. The IRB approval number is **FMH-03-2021-IRB-881-M**. The research adhered to the conditions outlined by the IRB, ensuring compliance with ethical standards.

If the nature of the study or the research question is changed, fresh ethical clearance will be required.

## Consent

Informed consent was obtained from all participants prior to their inclusion in the study. Participants were provided with detailed information about the study’s purpose and procedures. They were assured of their right to withdraw from the study at any time without any negative consequences. All collected data were anonymized and handled confidentially to protect participants’ privacy.

## Disclosure

All authors have read and agreed to the published version of the manuscript

## Conflicts of Interest

The authors declare no conflicts of interest.

## Author Contributions

Conceptualization, S.N.M.; methodology, S.N.M., M.I., S.E., K.M., S.I., and S.A.; validation, S.N.M., M.I., S.E., K.M., S.I., and S.A.; data curation, S.N.M., M.I., and S.E.; formal analysis, S.N.M., M.I., S.I., N.Z., and S.A.; investigation, S.N.M., M.I., and S.E.; writing–original draft preparation, S.N.M., M.I., S.E., K.M., S.I., and S.A.; writing–review and editing, S.N.M., M.I., S.E., K.M., S.I., N.Z., and S.A.; supervision, S.N.M. and S.E.

## Funding

No funding from any funding organizations within the public, private, commercial, or nonprofit organizations was received to complete this research.

## Data Availability

The data that support the findings of this study are available from the corresponding author upon reasonable request from Dr Salima Naveed Manji at dr.salimanaveed@gmail.com.
